# Emergency critical care: a blind spot in the upstream phase of critical illness

**DOI:** 10.1186/s13054-026-06031-8

**Published:** 2026-04-19

**Authors:** Philipp Kümpers, Alexandros Rovas, Richard Köhnke, Linus Pinkernell, Michael Reindl, Mark Michael, Michael Bernhard

**Affiliations:** 1 Department of Medicine D, General Internal and Emergency Medicine, University Hospital Münster, Albert-Schweitzer-Campus 1, 48149 Münster, Germany; 2 Department of Acute and Emergency Medicine, Ameos Klinikum St. Clemens, Wilhelmstr. 34, 46145 Oberhausen, Germany; 3 Department of Emergency Medicine, Heinrich-Heine-University, University Hospital Düsseldorf, Moorenstr. 5, 40225 Düsseldorf, Germany

**Keywords:** Emergency critical care, Critical care delivery, Measurability, Clinical decision-making, Early stabilization, Patient flow, Boarding, Clinical trajectory, Dynamic assessment

## Abstract

**Background:**

An increasing proportion of critically ill patients receive prolonged, high-acuity care in the emergency department (ED) before admission to the intensive care unit. Despite its clinical relevance, this early phase of care remains poorly characterized and difficult to capture using existing metrics.

**Main body:**

This Perspective argues that this early phase of critical illness represents a distinct but underrecognized component of the critical care continuum, commonly referred to as emergency critical care (ECC). Rather than being defined by location or specialty, ECC is characterized by time-critical decision-making, dynamic trajectory, and evolving care demands. A major challenge in studying this phase is its limited measurability within current frameworks. We therefore propose a pragmatic conceptualization of ECC based on three complementary dimensions: disease severity, clinical care intensity, and therapeutic organ support. Together, these dimensions describe key aspects of early critical illness and provide a pragmatic basis for translating ECC dimensions into observable clinical and administrative variables suitable for research, governance, and system evaluation without imposing rigid definitions or thresholds.

**Conclusion:**

Conceptualizing ECC along clinically meaningful and observable dimensions offers a practical way to improve its visibility in research and system evaluation. Recognizing ECC as a spectrum of critical care delivery rather than a binary state may provide a pragmatic basis for describing and studying early critical illness.

## Background

### The invisible phase of critical care

Modern intensive care medicine has achieved substantial advances in the management of organ failure, sepsis, and complex critical illness. At the same time, an increasing proportion of critically ill patients receive prolonged, high-acuity treatment *before* admission to an intensive care unit (ICU). Emergency departments (EDs) now routinely deliver advanced monitoring, organ support, and complex decision-making while patients await definitive allocation or ICU transfer [1, 2].

Despite its clinical relevance, this phase of care remains poorly defined, rarely conceptualized as part of the critical illness trajectory, and inconsistently reflected in governance and research. Yet decisions made during this early phase frequently influence subsequent resource utilization, ICU length of stay, and patient outcomes [3–10]. This *Perspective* argues that this early, high-acuity phase of care represents a major blind spot in the modern critical care continuum and proposes a pragmatic dimensional framework that allows ECC to be described independently of specific organizational models or consensus definitions.

## Main text

### Emergency critical care: widely practiced, rarely defined

Across healthcare systems worldwide, critically ill patients frequently remain in the ED for extended periods after the need for intensive care has been identified [1, 2, 11]. During this time, emergency teams initiate and maintain invasive ventilation, vasoactive therapy, advanced diagnostics, and continuous physiological monitoring. In many systems, this care is initiated in resuscitation bays or other designated high-acuity areas of the ED, but is frequently prolonged as critically ill patients remain in the ED while awaiting definitive disposition. Functionally, much of this care is indistinguishable from early ICU management, yet it is rarely described or conceptualized as such [12].

Instead, the prolonged management of critically ill patients in the ED is often framed as a temporary or suboptimal state—primarily a consequence of limited ICU capacity—rather than as a legitimate and structured component of critical illness management. This invisibility does not reflect a lack of expertise. Emergency physicians (EPs), who increasingly have formal training in critical care medicine [13, 14], routinely deliver complex, high-acuity care under demanding conditions; the gap is conceptual rather than clinical.

For the purpose of this discussion, we refer to this early, high-acuity phase of care as emergency critical care (ECC), a term that has increasingly been used in the contemporary literature to describe this phase of critical illness management in the ED [12, 15, 16].

### The blind spot at the ED–ICU interface

The limited conceptual recognition of ECC creates a blind spot at the interface between emergency medicine (EM) and intensive care. Responsibility for patients in this phase is often ill-defined, resulting in variability in practice and potential safety risks. When ECC remains poorly characterized, accountability for monitoring quality of care, staffing models, and outcome evaluation may become fragmented between EM and intensive care services. Making ECC analytically visible may therefore represent an important prerequisite for developing shared governance structures for critically ill patients during this transitional phase.

Historically, reporting and quality metrics typically focus either on ED *throughput* or ICU *outcomes*, leaving the transition phase largely unexamined. As case complexity has increased and ICU admission thresholds have risen, the early management of critical illness has shifted upstream, shaping ICU utilization and outcomes well before admission [17–19]. However, critical care frameworks have not fully adapted to this shift. As a result, ECC remains largely invisible in clinical governance, training pathways, and research agendas.

However, ECC should not be understood as an extension of intensive care medicine into the ED, as is sometimes implied by concepts such as ICU outreach or “critical care without walls,” which typically describe intensivist-led support for deteriorating ward patients [20, 21]. Nor should ECC be defined by the performance of individual critical care procedures or interventions. Instead, it reflects the ongoing management of physiological instability requiring sustained monitoring, intervention, and trajectory-guided clinical decision-making to inform early de-escalation, or definitive ICU allocation. Published evaluations from several centers suggest that ECC initiatives can shorten time to advanced care, reduce short-stay ICU admissions, and preserve downstream ICU capacity [4–7].

Within this context, ECC can be characterized as:

- **Temporally bounded**, with duration varying depending on local structures and resource constraints,
- **Rooted in emergency medicine**, while applying principles of critical care management,
- **Focused on resuscitation**,** early stabilization**,** and trajectory-guided clinical decision-making**, rather than definitive ICU-level care,
- **Operationally upstream of the ICU**, yet integral to the overall continuum of critical care.

In this sense, ECC functions as an upstream filter, preserving ICU capacity for patients with sustained or progressive organ failure and constituting a core element of the critical care continuum.

### Making emergency critical care measurable

A central challenge in studying ECC lies in the fact that ECC cannot be reliably classified as a binary clinical state. Unlike specific disease entities or clearly defined treatment locations, ECC does not represent *a discrete category* that can be labelled as present or absent. Critically ill patients may transition gradually between conventional emergency care and sustained high-acuity management, with intensity of monitoring, reassessment, and organ support evolving over time. Attempts to define ECC using fixed criteria or definitions therefore remain inherently limited.

Accordingly, we propose a pragmatic framework in which ECC can be described along three complementary, clinically observable *dimensions* that can be approximated using routinely available data:

- **Disease severity** (how sick the patient is),
- **Clinical care intensity** (how much care the patient requires),
- **Therapeutic organ support** (what organ support is being provided).

While related, these *dimensions* are not interchangeable and do not necessarily correlate: patients may exhibit high disease severity with limited organ support, or require intensive monitoring and care despite only moderate physiological derangement.

These dimensions reflect how care is delivered in practice rather than where it is delivered and are therefore applicable across different healthcare systems and organizational models (Table 1). Conceptually, they define a multidimensional space in which episodes of ECC can be positioned and compared over time.

**Table 1:** Conceptual dimensions for describing and quantifying emergency critical care

Dimension	Indicators (examples)	Conceptual meaning
**Disease severity**	Hypoxemia, hypotension, altered level of consciousness, metabolic derangement, lactate elevation, early organ dysfunction	Baseline severity of illness and short-term physiological instability, including response to initial stabilizing interventions
**Clinical care intensity**	Frequency of clinical reassessment, continuous physiological monitoring, serial laboratory testing, diagnostic intensity (e.g. advanced imaging, endoscopy), invasive access and procedures, nursing workload and continuous bedside care	Intensity of clinical care over time, reflecting diagnostic uncertainty, time pressure, and sustained medical and nursing effort
**Therapeutic organ support**	Vasopressor therapy, high-flow oxygen or non-invasive ventilation, invasive mechanical ventilation, airway management, hemostatic resuscitation, extracorporeal life support	Degree and duration of active organ support during early critical illness, reflecting immediate physiological need and evolving escalation or de-escalation decisions

A pragmatic starting point for empirical investigation of ECC may therefore be the systematic capture of the proposed ECC dimensions in all ED patients require ongoing monitoring or high-acuity therapy beyond what would typically allow immediate ward admission.

Within this population, disease severity, clinical care intensity, and therapeutic organ support could be systematically recorded (as outlined below), enabling a structured characterization of early critical care delivery.

Such an approach would allow the identification of recurring patterns of care. Rather than analyzing ECC as a single entity, these patterns may be clustered and conceptualized as distinct ECC *prototypes*, which could then be examined with respect to patient trajectories, resource utilization, and outcomes.

**Fig. 1:**
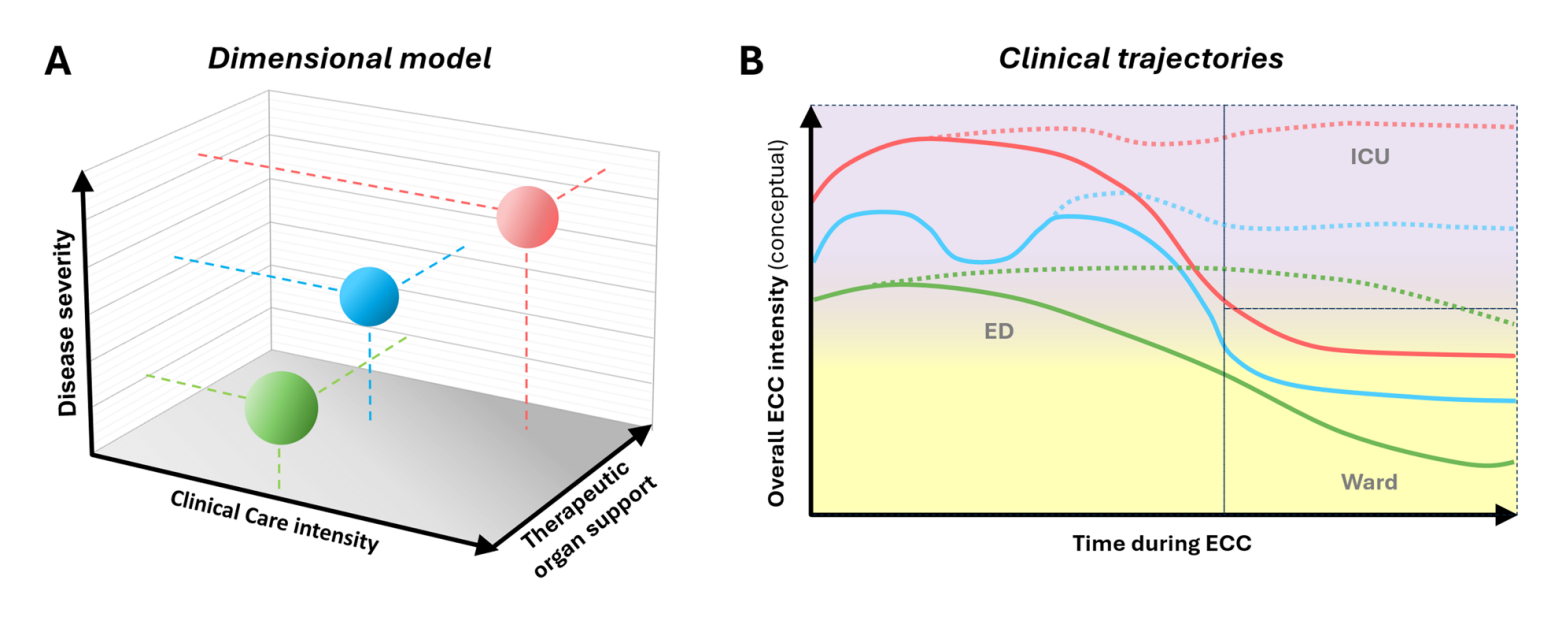
Dimensional and trajectory-based representation of emergency critical care (ECC). (**A**) Conceptual representation of ECC as a three-dimensional space defined by disease severity, clinical care intensity, and therapeutic organ support. Episodes of care can be positioned within this space based on clinically observable and measurable characteristics. Illustrative examples include symptomatic hyponatremia (green), hypertensive pulmonary edema (blue), and septic shock due to obstructive uropathy (red), representing different constellations across dimensions, with hyponatremia characterized by high clinical care intensity with limited organ support, hypertensive pulmonary edema by combined physiological instability and transient organ support, and urosepsis with obstructive uropathy by high disease severity requiring sustained organ support and intensive care delivery. These examples are illustrative and do not imply specific management pathways or disposition decisions. (**B**) Schematic trajectories of overall ECC intensity over time, reflecting the combined contribution of disease severity, clinical care intensity, and therapeutic organ support. Changes in trajectory reflect dynamic reassessment with escalation or de-escalation of care [7–9, 12]

Figure 1 illustrates this conceptual framework using three illustrative examples of common ECC prototypes positioned within the multidimensional space (Panel A) and their corresponding clinical trajectories over time (Panel B). While some of these prototypes are readily observable in routine clinical practice [22–24], their relationship to patient trajectories, resource utilization, and broader system-level effects has not yet been systematically studied, representing an important area for future investigation.

A prerequisite for such investigation, however, is the ability to translate these conceptual dimensions into measurable clinical variables. Importantly, many elements reflected in the proposed framework are already captured within routine digital clinical documentation, although they are often not readily available in a structured or aggregated form and may require additional processing or implementation of dedicated metrics for systematic use.

*Disease severity* may be approximated through repeated assessment of early warning scores (e.g. National Early Warning Score 2) or adapted organ dysfunction metrics (e.g. Sequential Organ Failure Assessment) [25, 26], allowing physiologic instability to be represented longitudinally rather than at a single time point. Given the trajectory-based nature of ECC, such measurements require higher temporal resolution than traditional point-in-time scoring approaches.

*Clinical care intensity* may be approximated using concepts analogous to established intensive care workload instruments such as the Therapeutic Intervention Scoring System (TISS)-28 or the Nursing Activities Score, which quantify care complexity based on performed interventions [27]. Applied to the ED, this dimension reflects the frequency of reassessment, monitoring demands, diagnostic and therapeutic workload, and continuous medical and nursing attention.

*Therapeutic organ support* represents a particularly tractable dimension for operationalization, as many life-sustaining interventions delivered during ECC—such as respiratory support, vasoactive therapy, or invasive monitoring—are routinely documented and can be translated into measurable variables by capturing their presence, intensity, and duration.

Building on these variables, structured capture and longitudinal aggregation of clinical data across ECC dimensions may enable reporting of ECC delivery in a manner conceptually analogous to established ICU severity and workload metrics. Such approaches could provide the foundation for composite measures of ECC intensity and for identifying recurring patterns of early critical care delivery.

Importantly, improved characterization of ECC should not be equated with ICU-level reimbursement. Rather, its primary value lies in enabling more transparent description, evaluation, and governance of a phase of care that has historically remained difficult to quantify, and in supporting future research on how early critical care delivery influences downstream resource utilization and patient outcomes [4, 5, 7–9].

## Conclusions

ECC represents a clinically important yet insufficiently characterized phase of critical illness. Although a substantial proportion of high-acuity care is delivered before ICU admission, this phase remains poorly captured by existing critical care frameworks and metrics.

By conceptualizing ECC along dimensions of disease severity, clinical care intensity, and therapeutic organ support, this *Perspective* proposes a pragmatic way to describe and study early critical illness without imposing rigid definitions or thresholds. This dimensional approach provides a basis for examining how early clinical trajectories relate to downstream resource use and outcomes.

Recognizing ECC as a spectrum of critical care delivery rather than a discrete category may help improve the visibility of this phase within critical care research and system evaluation. Making this upstream phase analytically visible may also help focus clinical and organizational attention on a period of illness that has historically remained difficult to quantify.

## Data Availability

Not applicable.

## Abbreviations

EM: Emergency medicine
ED: Emergency department
ICU: Intensive care unit
ECC: Emergency Critical Care
EP: Emergency physician
